# Serum CA125 is a predictive marker for breast cancer outcomes and correlates with molecular subtypes

**DOI:** 10.18632/oncotarget.19246

**Published:** 2017-07-12

**Authors:** Cheng Fang, Yue Cao, Xiaoping Liu, Xian-Tao Zeng, Yirong Li

**Affiliations:** ^1^ Department of Laboratory Medicine, Zhongnan Hospital of Wuhan University, Wuhan, China; ^2^ Center for Evidence-Based and Translation Medicine, Zhongnan Hospital of Wuhan University, Wuhan, China

**Keywords:** serum tumor markers, CA125, breast cancer, clinicopathological features, molecular subtype

## Abstract

Detection of serum tumor markers has been developed as a non-invasive tool to assess treatment efficiency in different types of cancer. This study aims to investigate the role of preoperative serum tumor markers (CEA, CA125 and CA15-3) in the management of breast cancer, and their relationships with patients’ clinicopathological parameters as well as different molecular subtypes. Altogether, 151 patients with invasive breast cancer and 180 control subjects with benign breast diseases were enrolled in this study. In the present study, preoperative serum levels of CEA, CA125 and CA15-3 were significantly higher in patients with breast cancer than controls subjects. Moreover, late-stage cancer patients exhibited significantly higher levels of CEA, CA125 and CA15-3 compared with early-stage ones. Statistical analysis indicated that elevated CA125 and CA15-3 levels were obviously related to patients with larger tumor diameter (>5cm) and lymph node metastasis. Furthermore, our results showed that the preoperative serum levels of CA125 exhibited statistical differences among various molecular subtypes, with the most frequent elevations occurring in the triple-negative tumors. In summary, our study indicated that the preoperative serum levels of CEA, CA125 and CA15-3 might be more efficient for monitoring advanced tumors than early diagnosis. High preoperative CA125 levels may reflect tumor burden and are associated with aggressive molecular subtype, suggesting that it can be used to predict poor outcome and prognosis of breast cancer patients.

## INTRODUCTION

Breast cancer is the most common malignancy that affects females, accounting for 23% of all cancer deaths worldwide [1]. It has been reported that an estimated 269,000 new cases of breast cancer were diagnosed with nearly 70,000 cancer deaths in China in 2015, and its incidence has been steadily increasing [2]. The treatment of this multifactorial disease is closely related to patients’ clinicopathological factors, such as tumor size, lymph node involvement, hormone receptor status and HER-2 status. For practical purposes, biological subtyping by use of immunohistochemical surrogate panel of biomarkers (ie, ER, PR, HER-2 and Ki-67) are similar to intrinsic subtypes and represent a convenient approximation. Breast cancer is considered to be a heterogeneous disease and mainly classified into four molecular subtypes, including Luminal A, Luminal B, HER-2/neu and triple-negative [3–4]. Currently, individualized treatment based on molecular subtypes has become an important issue in breast cancer research.

Quantitative variations of serum tumor markers have been developed as non-invasive tools for the assessment of treatment efficiency in human malignancies [5–6]. In breast cancer, carcinoembryonic antigen (CEA), cancer antigen 125 (CA125) and cancer antigen 15-3 (CA15-3) are the most widely used serum tumor markers in clinical routine, although their usefulness remains controversial [5, 7]. CEA is a glycoprotein, relevant for cell adhesion, and was the first tumor antigen that has been studied [8]. Serum CEA levels can rise when inflammation or cancer involves endodermal tissues, such as gastrointestinal, pancreatic and breast tissues [9]. CA125 is proposed as a serum biomarker for ovarian cancer, but elevated levels have been observed in up to 84% of metastatic breast patients [10], and correlated with the metastasis-associated burden in pancreatic cancer [6]. CA15-3 is routinely used in monitoring therapy and predicting recurrences in patients affected by breast cancer [11]. The serum levels of CEA, CA125 and CA15-3 were demonstrated to be of great value in the management of patients with breast cancer, and could serve as predictive indicators and for monitoring the course of disease [12].

Previous studies have been conducted to quan-titatively evaluate the serum level of above tumor markers in breast cancer patients, and correlations between tumor marker elevations and patients’ ethnicity, clinical tumor stages and tumor burden were investigated [13–15]. Due to inconsistent results, their clinicopathological significance remains to be elaborated. Furthermore, little is known about the correlation between these markers and breast cancer subtypes. Therefore, we carried out a retrospective study to determine the clinicopathological significance of preoperative serum tumor markers (CEA, CA125 and CA15-3) in Chinese breast cancer patients as well as the relevance of these markers among different breast cancer subtypes.

## RESULTS

### Subject characteristics

According to the inclusion criteria, 151 invasive breast cancer cases and 180 control subjects who underwent breast surgery at Zhongnan Hospital of Wuhan University were eventually enrolled. The mean age was 49.9±10.9 and 48.0±8.0 years in cancer and control subjects, respectively. The clinicopathological characteristics of the patients were extracted from medical records. A majority of cancer patients (86.1%) were diagnosed as invasive ductal carcinoma (IDC). Among all cases, stage I, II and III breast cancer accounted for 13.2%, 56.3% and 30.5%, respectively. Out of 151 cases, 26 patients (17.2%) were classified as Luminal A, 79 patients (52.3%) as Luminal B, 22 patients (14.6%) as HER-2/neu and 24 patients (15.9%) as triple-negative (Table 1). Control subjects with breast benign diseases were also pathologically confirmed, including mammary hyperplasia and fibroadenoma.

**Table 1:** General characteristics of study population

Characteristics	N	Percentage
Age (years)		
<50	78	51.7%
≥50	73	48.3%
Size		
T1	37	24.5%
T2	93	61.6%
≥T3	21	13.9.%
Node status		
N0	69	45.7%
≥N1	82	54.3%
TNM stage		
I	20	13.2%
II	85	56.3%
III	46	30.5%
Pathological type		
Ductal	130	86.1%
Others	21	13.9%
Molecular subtype		
Luminal A	26	17.2%
Luminal B	79	52.3%
HER-2/neu	22	14.6%
Triple-negative	24	15.9%

### Main results

**Figure 1:**
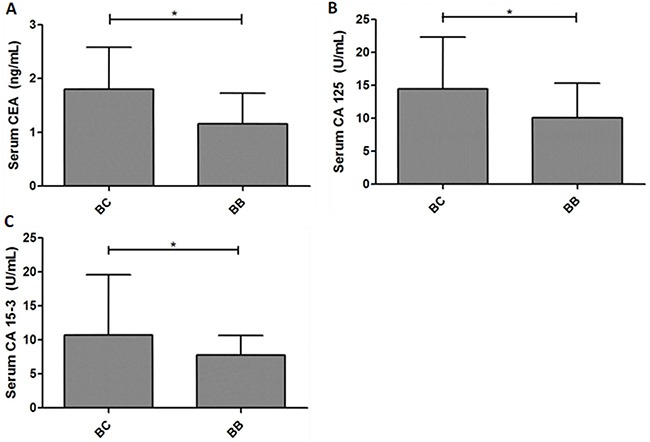
The expression levels of serum tumor markers discriminate between breast cancer patients and control subjects The preoperative serum levels of CEA, CA125 and CA153 were significantly higher in patients with breast cancer than benign disease. *P<0.05.

As shown in Figure 1, the preoperative serum levels of CEA, CA125 and CA15-3 were significantly higher in patients with breast cancer than control subjects (*P*<0.05). Elevated serum levels (above cut-off values) of CEA, CA125 and CA15-3 were identified in 13 (8.6%), 21 (13.9%) and 14 (9.3%) breast cancer cases, but not observed in breast benign diseases. Among them, 17 (16.2%) patients with early cancer stage had tumor marker concentration above cut-off. The present data demonstrated the preoperative serum levels of CEA, CA125 and CA15-3 discriminated between patients with breast cancer and benign diseases. However, only a small percentage of cancer patients had preoperative levels of serum tumor markers above cut-off, confirming low sensitivity of these markers in breast cancer diagnosis.

The relationship between serum levels of tumor markers and clinicopathological factors of breast cancer patients were displayed in Table 2. In our study, late-stage cancer patients exhibited significantly higher levels of CEA, CA125 and CA15-3 compared with early-stage ones, suggesting that the serum levels of these tumor markers might be more efficient for monitoring advanced tumors than early diagnosis. Statistical analysis indicated that both elevated CA125 and CA15-3 levels were obviously related to patients with larger tumor diameter (>5cm) and lymph node metastasis.

**Table 2:** Relationship between serum marker levels and clinicopathogical features of breast cancer

Characteristic	N	CEA (ng/ml)		CA125 (U/mL)		CA153 (U/mL)	
		Median (P25, P75)	*P*-value	Median (P25, P75)	*P*-value	Median (P25, P75)	*P*-value
**Size**			0.26		0.03		<0.01
T1	37	1.72 (1.38, 2.53)		13.80 (8.60, 21.01)		9.60 (6.55, 20.09)	
T2	93	1.80 (1.22, 2.52)		13.72 (7.68, 21.73)		10.51 (7.68, 16.41)	
≥T3	21	2.30 (1.17, 6.58)		19.18 (12.98, 92.81)		19.29 (11.88,50.92)	
**Node status**			0.63		0.01		0.03
N0	69	1.60 (1.08, 2.36)		10.66 (7.72, 18.73)		9.73 (7.41, 15.70)	
≥N1	82	1.94 (1.31, 2.93)		15.68 (9.06, 27.45)		13.10 (7.70, 23.63)	
**TNM stage**			0.01		0.01		<0.01
I/II	105	2.24 (1.18, 2.26)		24.16 (8.00, 19.98)		12.87 (7.14,16.11)	
III	46	8.16 (1.32, 3.28)		56.38 (10.51, 31.00)		26.75 (9.23, 29.03)	
**Molecular subtype**			0.48		<0.01		0.26
Luminal A	26	1.64 (0.98, 2.70)		17.45 (10.44, 22.81)		9.74 (7.15, 19.36)	
Luminal B	79	1.82 (1.09, 2.56)		10.66 (7.26, 18.30)		10.65 (7.89, 17.56)	
HER-2/neu	22	1.93 (1.43, 4.24)		12.89 (9.76, 19.75)		10.88 (6.32, 16.51)	
Triple-negative	24	1.50 (1.24, 2.26)		46.46 (14.60, 82.19)		14.83 (8.39, 33.76)	

P25, the 25th percentile; P75, the 75th percentile.

**Figure 2:**
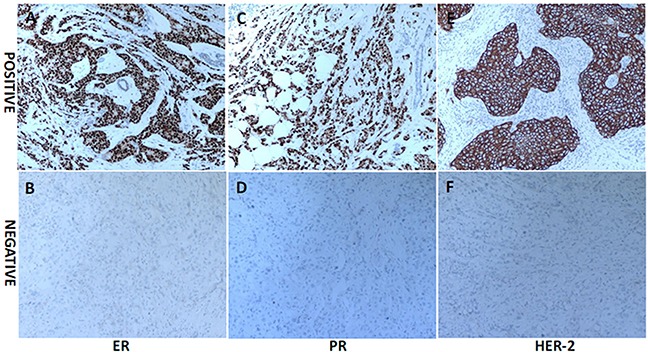
The representative immunohistochemistry (IHC) results of ER, PR and HER-2 expression in breast cancer (original magnification ×100) Tumors with >1% nuclear-stained cells were considered positive for the ER and PR. HER-2 positivity was evaluated as membrane staining of invasive tumor cells and scored by 3+.

**Figure 3:**
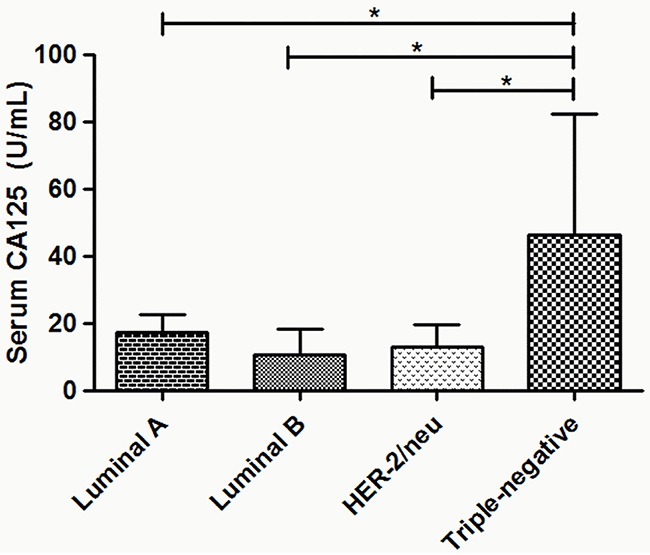
The association between preoperative serum CA125 levels and molecular subtypes in breast cancer patients The elevated serum levels of CA125 were more frequently observed in triple-negative patients compared with Luminal A, Luminal B and HER-2/neu patients. *P<0.05.

The representative immunohistochemistry results of ER, PR and HER-2 expression were presented in Figure 2, different molecular subtypes were then identified. Interestingly, our results showed that the serum levels of CA125 exhibited statistical differences among various molecular subtypes. Further analysis indicated that elevated CA125 was more frequently observed in triple-negative patients compared with Luminal A, Luminal B and HER-2/neu patients (*P*<0.01, Figure 3).

## DISCUSSION

In the present study, the preoperative serum levels of CEA, CA125 and CA15-3 were significantly higher in patients with breast cancer than control subjects. Elevated serum levels of CEA, CA125 and CA15-3 were identified in 13 (8.6%), 21 (13.9%) and 14 (9.3%) breast cancer cases, which are similar to those observed in other studies [16–17]. Despite difference between two groups is statistically significant, this difference is of limited clinical significance due to: 1) within control group and within the gross majority of breast cancer patients, measured levels of serum tumor markers were bellow selected cut off values, and 2) there is a huge overlap in measured levels of all three studied markers between control and cancer patients.

In our study, patients with late-stage breast cancer exhibited significantly higher levels of CEA, CA125 and CA15-3 compared with early-stage ones, suggesting that the serum levels of these tumor markers might be more efficient for monitoring advanced tumors than early diagnosis. With the improvement of screening techniques for tumor detection, the detection rate of breast cancer has been steadily increasing in the last two decades, and early breast cancer accounted for a large proportion [14, 18]. As expected, with the increase of early breast cancer patients in study population, decreased prevalence of abnormal serum CEA, CA125 and CA15-3 was shown, leading to limited sensitivity in early diagnosis of breast cancer. However, this does not mean that their clinical value is also low. Since elevated levels of these markers are related to late tumor stage, poor outcome may be predicted, and more comprehensive therapy for the patients may be developed. Moreover, our results showed that preoperative serum levels of CA125 and CA15-3 were obviously associated with tumor burden indicators, including tumor size and axillary lymph node status.

It is known that hormone receptor (ER and PR) and HER-2 status plays a prominent role in the current molecular classification of breast cancer and can serve as remarkable prognostic factors [19–20]. However, the association between serum tumor markers and breast cancer subtype is not well established. The study by Shao et al. showed a strong correlation of elevated CA 15-3 levels with ER positivity [17], whereas Moazzey et al. reported that no statistical difference existed in serum CEA and CA15-3 among different molecular subtype groups [15]. In this study, we did not find any difference between serum CEA and CA15-3 levels among different subtypes. However, our result showed that serum CA125 was significantly greater in patients with triple-negative tumors than in Luminal A, Luminal B and HER-2/neu tumors, which may be explained in part by the different biological behaviors of different molecular subtypes. As a highly heterogeneous disease, individualized treatment based on molecular subtype has become an important issue in breast cancer research. Commonly, Luminal type tumors are sensitive to endocrine therapy, and targeted therapy is effective for HER-2/neu tumors. Triple negative breast cancer is reported to have poor prognosis due to the lack of targeting therapy and the biology of the tumor itself [21–22]. Hence, elevated CA125 levels may be one factor that predicts a poor prognosis.

There are some limitations should be acknow-ledged. First, this is a retrospective study, the biases related to incomplete medical records and physician decisions about when and in what circumstances they request tumor markers may confound the results. Second, it is possible that differences in some confou-nding factors, such as age, menopause status, body mass index (BMI), lifestyle and environment may interfere with tumor marker levels [13]. Third, as a heterogeneous disease, breast cancer may require combining multiple biomarkers to allow the detection of different subtypes. Additionally, the main caveat of this study is related to its small sample size, which may affect the results.

Nevertheless, this study contains a number of strengths. Importantly, the inclusion criteria for breast patients were rigorous and preoperative serum levels of tumor markers were reported predated the molecular subtyping of breast cancer. Thus, physician bias about the utility of measuring tumor markers in various breast cancer subtypes was not an issue. Moreover, the serum levels of CEA, CA125 and CA15-3 were measured using automatic chemiluminescence immunoassay system, which is noninvasive and easy to perform. And its characteristics of good precision and reliability, makes its employment universal in clinical routine. Furthermore, our data may be helpful in trials that use tumor markers as therapeutic targets for novel interventions or surrogates for clinical benefit in patients with non-measurable diseases [23]. However, given the limitations elaborated above, multi-center prospective studies with larger sample sizes should be employed to confirm the role of these tumor markers in further research. In addition, we need to add some extra notes to propose a clinical score that involves clinical and biochemical/ molecular data in future study.

In summary, our study indicated that the preoperative serum levels of CEA, CA125 and CA15-3 discriminated between patients with invasive breast cancer and breast benign diseases. Additionally, the serum levels of above tumor markers might be more efficient for monitoring advanced tumors than early diagnosis. High preoperative CA125 levels may reflect tumor burden and are associated with aggressive molecular subtype, suggesting that it can be used to predict poor outcome and prognosis of breast cancer patients. Summarizing our results, we recommend that patients with elevated CEA, CA125 and CA153 levels suggestive of breast cancer receive subsequent examinations or clinical interventions.

## MATERIALS AND METHODS

### Study population and inclusion criteria

A total of 331 female patients who underwent breast surgery at Zhongnan Hospital of Wuhan University between June 2012 and December 2015 were included into this study. A retrospective analysis of the clinicopathological data collected from 151 breast cancer patients was conducted. Inclusion criteria for these patients were: (1) being pathologically confirmed as invasive breast cancer patients; (2) with no history of cancer; and (3) with complete clinicopathological data, including age, tumor size, clinical stage, axillary lymph node status, expression of ER, PR and Ki-67. Meanwhile, 180 age-matched control subjects were also selected. These control subjects were pathologically confirmed as breast benign diseases, including mammary hyperplasia and fibroadenoma. This study was approved by the ethics committee of Zhongnan Hospital of Wuhan University, in accordance with the Declaration of Helsinki. Written informed consents were obtained from all patients.

### Measurement of serum CEA, CA125 and CA15-3 levels

Peripheral blood samples were collected from all patients before surgery. Then serum was separated by centrifugation (2500 rpm for 10 min) and kept at −20°C for later analysis. We measured the serum levels of CEA, CA125 and CA15-3 using automatic chemiluminescence immunoassay system (Abbott i-2000, Abbott, USA). A cut-off limit of 5 ng/mL (CEA), 35 U/mL (CA125) and 31.3 U/mL (CA15-3) was used as recommended by the manufacturer.

### Immunohistochemical evaluation

Tumor tissues were collected at the surgery and immunohistochemistry (IHC) method was then used to detect the expression of ER, PR, HER-2 and Ki-67. Tumors with >1% nuclear-stained cells were considered positive for the ER and PR. HER-2 positivity was indicated by a 3+ score from the immunohistochemical evaluation. A cut-off point of 14% was used for Ki-67 staining. According to above detection results, we defined the molecular subtypes as follows [24]: Luminal A (ER and/or PR positive, HER-2 negative and Ki-67<14%); Luminal B (ER and/or PR positive, HER-2 positive; ER and/or PR positive, HER-2 negative and Ki-67≥14%); HER-2/neu (ER and PR negative but HER-2 positive) and triple-negative (ER, PR and HER-2 negative).

### Clinical stage

According to standard criteria based on data of TNM (Tumor, Nodes and Metastases) and American Joint Committee on cancer (AJCC) staging system [25], stage ≤II was assigned as early-stage and stage >II was assigned as late-stage.

### Statistical analysis

All statistical analyses were performed with SPSS 17.0 software. As the data of serum tumor marker levels did not fit a Gaussian distribution, non-parametric tests (the Mann-Whitney test for 2 independent groups, and the Kruskal-Wallis test for 3 independent groups) were applied. The reported data were characterized by their median and quartiles (the 25th to 75th percentile). A value of *P*<0.05 was considered to be statistically significant.
